# Linear glandular trichomes of *Helianthus* (Asteraceae): morphology, localization, metabolite activity and occurrence

**DOI:** 10.1093/aobpla/plt028

**Published:** 2013-06-07

**Authors:** Anna-Katharina Aschenbrenner, Silke Horakh, Otmar Spring

**Affiliations:** Institute of Botany, University of Hohenheim, Garbenstraße 30, 70593 Stuttgart, Germany

**Keywords:** Glandular trichome, Helianthinae, *Helianthus*, multicellular, scanning electron microscopy, sesquiterpenes, sunflower, uniseriate.

## Abstract

Capitate glandular trichomes of sunflower are well investigated, but detailed studies are lacking for the linear glandular trichomes (LGT), a second type of physiologically active plant hair present on the surface of sunflowers. Light, fluorescence and scanning electron microscopy as well as histochemical staining were used to investigate the structure and metabolite deposition of LGT. Consisting of 6–11 linearly arranged cells, LGT were found on the surface of most plant organs of *Helianthus annuus*. They were associated with the leaf vascular system, and also occurred along petioles, stems and the abaxial surface of chaffy bracts, ray and disc florets. The highest density was found on the abaxial surface of phyllaries. Phenotypically similar LGT were common in all species of the genus, but also occurred in most other genera of the Helianthinae so far screened. Brownish and fluorescent metabolites of an as yet unknown chemical structure, together with terpenoids, were produced and stored in apical cells of LGT. The deposition of compounds gradually progressed from the tip cell to the basal cells of older trichomes. This process was accompanied by nucleus degradation in metabolite-accumulating cells. The localization of these trichomes on prominent plant parts of the apical bud and the capitulum combined with the accumulation of terpenoids and other as yet unknown compounds suggests a chemo-ecological function of the LGT in plant–insect or plant–herbivore interaction.

## Abstract (precis)

The plant surface of sunflower is covered with at least three types of trichomes, two of which have glandular nature and produce a rich spectrum of allelochemicals. While the development and metabolic activity of the biseriate multicelluar glands has been investigated intensively in the past, hardly any information was yet available on the uniseriate linear glands. The current study of this latter type of plant hairs provides first detailed results on: (i) the occurrence in *Helianthus* and related genera, (ii) the accumulation of sesquiterpenes and other metabolites, and (iii) a possible a function of the trichomes in plant insect interaction.

## Introduction

Plant trichomes are epidermal protuberances of highly variable shape, cytology and function (Uphof 1962; Johnson 1975; Spring 2000). More than 300 types of plant trichomes have been described (Wagner 1991). They occur on different surfaces of almost all angiosperms and may alter the boundary layer above the leaf surface, contribute to light piping, protect against temperature stress, reduce water loss in transpiration or serve as the absorbing surface in roots.

Besides metabolically inactive or so-called non-glandular trichomes (NGT), biosynthetically active glandular tri-chomes (GT) also exist. They sequester or store plant metabolites that are often characteristic for specific taxonomic groups [e.g. monoterpenes in Lamiaceae, sesquiterpene lactones (STL) in Asteraceae]. The storage of bioactive compounds in GT is frequently associated with protection against herbivores and pathogens (Ågren and Schemske 1994), but evaporation of volatile trichome metabolites also occurs and may have numerous physiological and ecological functions (McCaskill and Croteau 1995).

The Asteraceae family with its ∼25 000 species (Panero and Funk 2008) in over 1100 genera is rich in different trichome types. Castro *et al*. (1997) reviewed the secretory structures in the leaves of 72 representative Asteracean species and found eight different types of trichomes, among them uniseriate (composed of one row of cells) and peltate types. Krak and Mráz (2008) used the trichome morphology for classification of the tribe Lactuceae (Asteraceae). Besides NGT, they also observed multiseriate (composed of two or more cell rows) GT with a multicellular head, uniseriate GT with a multiseriate base and a unicellular head, and bi- or multiseriate GT.

In Asteraceae, trichome studies focused on capitate glandular trichomes (CGT), which are common in many tribes of the family and are known to produce thousands of STL structures (Seaman 1982; see also reviews by Fraga 1987, 2011), sometimes in combination with flavonoids, acetophenones and other compounds (Spring 1991; Spring *et al*. 1994; Göpfert *et al*. 2005, 2006, 2009).

Previous studies have shown that the leaves of *Helianthus annuus* harbour two different types of GT, namely the above-described biseriate multicellular CGT and linear glandular trichomes (LGT) (Spring *et al*. 1992; Harr and Guggenheim 1995; Göpfert *et al*. 2010). In addition to their morphology, the two types of GT differ significantly in localization, development and metabolic chemistry. While the CGT have been reported from many taxa of Asteraceae (Kelsey *et al*. 1984; Spring and Buschmann 1996; Spring 2000), only two reports exist focusing on the LGT of sunflower: one described the occurrence of LGT on sunflower stems, leaf petioles and leaves (particularly on veins) (Göpfert *et al*. 2010) and the other showed the presence of bisabolene-type sesquiterpenes stored in LGT (Spring *et al*. 1992).

This study provides for the first time detailed results on the morphology, localization and metabolic activity of LGT in sunflower and their occurrence in related taxa.

## Methods

### Plant material

*Helianthus annuus* cv. Giganteus plants were grown under greenhouse conditions with an additional 16-h illumina-tion (330 μmol s^−1^ m^−2^) and a dark period of 8 h. Alter-natively they were grown in the field (Botanical Garden, University of Hohenheim, Stuttgart, Germany).

Other *Helianthus* species used as fresh material were cultivated in the field or were investigated in desiccated form by means of herbarium specimens (HOH, collected by O. Spring, Stuttgart, Germany). All species used are listed in Table 1.

**Table 1.** Species of the tribe Heliantheae screened for LGT (+, present; −, not detected) using fresh plant material (FPM) from field-cultured plants or herbarium specimens (HOH).

Species	Source	LGT	Species	Source	LGT
Helianthus					
*H. annuus*	FPM	+	*H. neglectus*	HOH (Schilling F84-1)	+
*H. angustifolius*	HOH (OS 54)	+	*H. nutallii*	HOH	+
*H. atrorubens*	HOH (Schilling H80-15)	+	*H. occidentalis*	HOH	+
*H. ciliaris*	HOH (Jackson H6-116-1)	+	*H. pauciflorus*	HOH	+
*H. debilis* ssp*. cucumerifolius*	HOH	+	*H. petiolaris* ssp*. fallax*	HOH (G.S. 12a)	+
*H. debilis* ssp*. tardiflorus*	HOH	+	*H. porteri*	HOH (Schilling 1986)	+
*H. decapetalus (2n)*	FPM	+	*H. praecox* ssp. *hirtus*	HOH (OS 81)	+
*H. eggertii*	HOH	+	*H. praecox* ssp*. praecox*	HOH (OS 83)	+
*H. floridanus*	HOH	+	*H. praecox* ssp*. runyonii*	HOH (OS 85)	+
*H. glaucophyllus*	HOH	+	*H. pumilus*	HOH	+
*H. gracilentus*	HOH	+	*H. radula*	HOH (Schilling 80-29)	+
*H. grosseserratus*	HOH (OS 35)	+	*H. resinosus*	HOH (Schilling 80-37)	+
*H. heterophyllus*	HOH (OS 58)	+	*H. salicifolius*	FPM	+
*H. hirsutus*	HOH	+	*H. schweinitzii*	HOH	+
*H. lacineatus*	HOH (F8435)	+	*H. silphioides*	HOH	+
*H. maximiliani*	HOH (OS 43)	+	*H. simulans*	HOH (OS 54)	+
*H. microcephalus*	HOH (OS4)	+	*H. strumosus*	HOH (OS 138)	+
*H. mollis*	HOH (OS 41)	+	*H. tuberosus*	FPM	+
*H. niveus* ssp*. niveus*	HOH (Schilling 10121)	+	*H.* × *laetiflorus*	FPM	+
*H. niveus* ssp*. tephrodes*	HOH (Schilling 10130)	+	*H.* × *multiflorus*	FPM	+
Other Helianthinae					
*Aldama aspilioides*	HOH (Da Costa 121)	−	*P. lehmannii*	HOH (Panero 1408)	+
*A. budleiformis*	HOH (Panero 587)	+	*Phoebanthus grandiflora*	HOH (OS 195)	+
*A. cordifolia*	HOH (OS 302)	−	*P. tenuifolius*	HOH (OS 204)	+
*A. flava*	HOH (Panero 529)	+	*Simsia amplexicaulis*	HOH (OS 326)	+
*A. robusta*	HOH (OS 355)	+	*S. lagascaeformis*	HOH (OS 313)	+
*Heliomeris hispida*	HOH (OS 260)	−	*Sidneya tenuifolia* (syn. *Viguiera stenoloba*)	HOH (OS 330)	−
*H. longifolia*	HOH (OS 291)	+	*Tithonia diversifolia*	HOH (Olivar Coya)	−
*H. multiflora*	HOH (OS 368)	−	*T. rotundifolia*	HOH (OS 25)	+
*H. soliceps*	HOH (Schilling 93-3)	−	*T. thurberi*	HOH (OS 295)	−
*Lagascea decipiens*	HOH (OS 297)	+	*T. tubaeformis*	HOH OS (323)	−
*Pappobolus decumbens*	HOH (Panero 1345)	+	*Viguiera dentate*	HOH (OS 331)	+
Other Heliantheae					
*Rudbeckia laciniata*	FPM	+	*Verbesina encelioides*	HOH (OS 252)	−
*R. triloba*	HOH (OS 15)	−	*V. longifolia*	HOH (OS 301)	−
			*V. rothrockii*	HOH (OS 306)	−

### Microscopic investigations

To examine the morphology and metabolic activity of LGT, conventional light microscopy (LM) and fluorescence microscopy (FM) were used. The localization on sunflower and the occurrence of trichomes in other taxa (Table 1) were checked using a stereo microscope. For closer investigation, single trichomes were collected from the surface or small pieces of epidermal stripes were prepared and investigated in water on microscopic slides. Micrographs were taken with a digital camera (Canon PowerShot A640) connected to an Axioplan microscope (Zeiss, Oberkochen, Germany). Different fluorescence filters (Zeiss), with different excitation wavelengths (nm), were used (Filter I: 05/395-440; excitation: 395–440 nm; beam splitter: 460 nm; emission: 470 nm; Filter II: 02/G365; excitation: 365 nm; beam splitter: 395 nm; emission: 420 nm). If necessary to create one completely focused image from several partially focused images, Helicon Focus 4.21 (Helicon Soft Ltd, Kharkov, Ukraine) was used.

To visualize the nuclei in the LGT of *H. annuus*, epidermal stripes were stained with trihydrochloride (Hoechst 33342, Life Technologies GmbH, Darmstadt, Germany) with additionally added 5% Tween 20 to reduce the surface tension and observed with the fluorescence microscope described above (Filter II).

A previous investigation has shown the occurrence of bisabolene-type sesquiterpenes in the LGT of *H. annuus* (Spring *et al*. 1992). To confirm the presence of terpenes in the LGT in general, histochemical staining with Nadi reagent (a mixture of 1-naphthol and *N*,*N*-dimethyl-*p*-phenylene diamine) for terpenes (Moura *et al*. 2005) and concentrated H_2_SO_4_ for sesquiterpenes (Cappelletti *et al*. 1986; Nikolakaki and Christodoulakis 2007) was performed as described in the literature using epidermal stripes.

For scanning electron microscopy (SEM), fresh leaf samples (0.5 cm²) were fixed with FAA fixative (formalin–glacial acetic acid −70% alcohol, 5 : 5 : 90). Before critical point drying (CPD), samples were washed twice with FAA for 15 min followed by dehydration with formaldehyde dimethyl acetal for 30 min according to Gersterberger and Leins (1978). The CPD was carried out with a CPD 020 (Balzers Union, Wiesbaden, Germany). The samples were coated with gold–palladium (80 : 20) using a sputter coater (SCD 040, Balzers Union) and investigated with a SEM DSM 640 (Zeiss, Oberkochen, Germany) at 5 kV.

### Statistical analysis

To determine the average number of cells and the length of LGT, 30 trichomes from the central leaf vein were ob-served, measured (AxioVision Rel. 4.8, Zeiss) and statistically analysed.

To analyse whether new trichomes are formed during leaf expansion, five plants were investigated at different time intervals. From each plant the central leaf vein of one of the primary leaves was measured and the number of LGT per cm of leaf vein (centrical) was counted (point in time T0). After 12 days (T12) of cultivation, the opposite primary leaf was used to repeat the same procedure. The data obtained were analysed statistically.

## Results

### LGT occurrence and morphology on H. annuus

**Figure 1.**
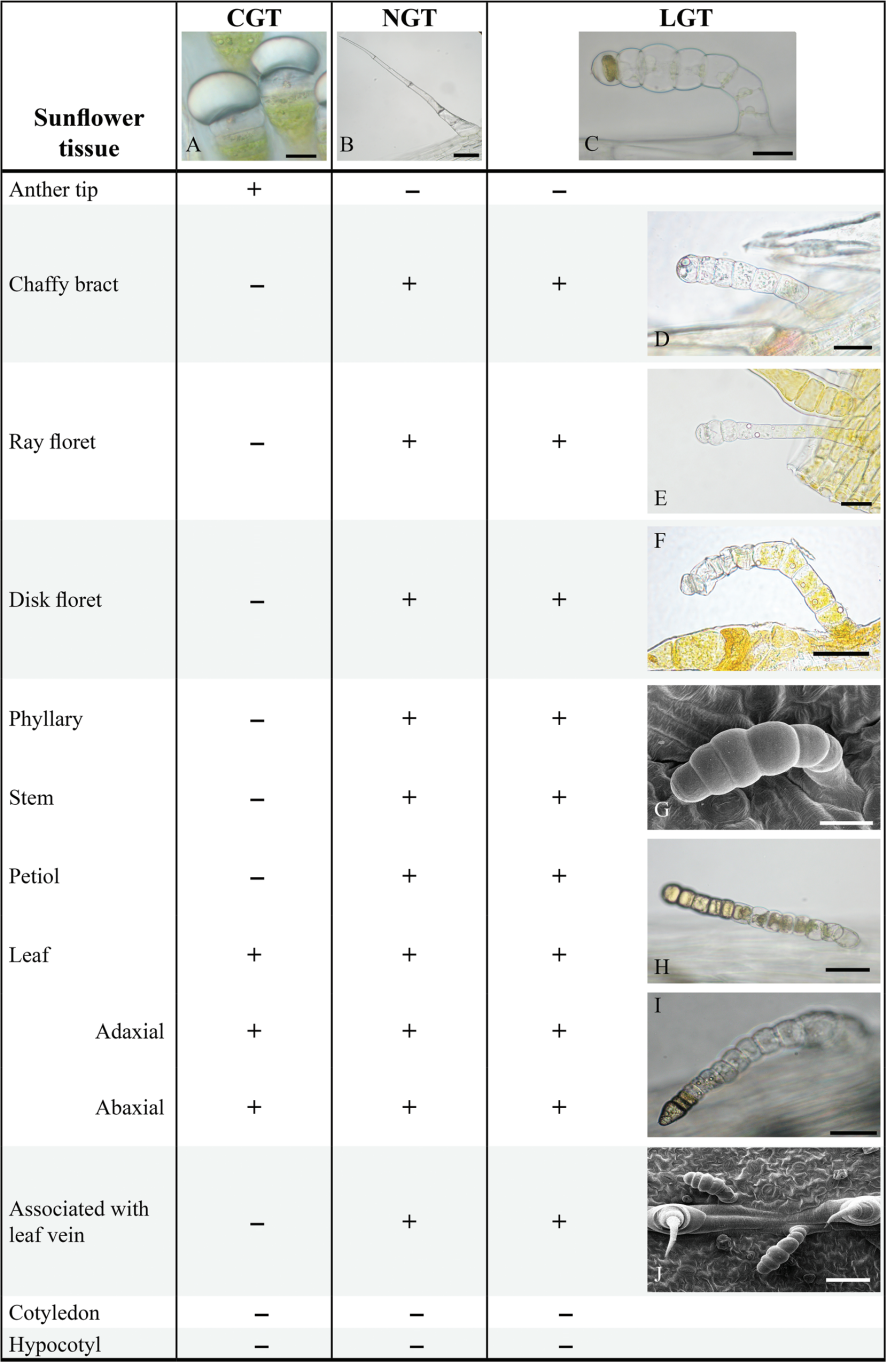
Occurrence and morphology of different types of trichomes on *H. annuus* tissues, documented by LM or SEM (G and J). CGT, capitate glandular trichomes (A, scale bar: 20 µm); NGT, non-glandular trichomes (B, scale bar: 100 µm); LGT, linear glandular trichomes (C–F, H and I, scale bar: 25 µm; G, scale bar: 20 µm; and J, scale bar: 50 µm). +, present; −, not detected. (G)–(I) exemplify the morphological characteristics on phyllaries, stems, petioles and the adaxial and abaxial leaf surfaces.

Linear glandular trichomes, often intermixed with NGT, were found on stems, leaf petioles and the abaxial surface of phyllaries, chaffy bracts, and ray and disc florets (Fig. 1). They were also observed on the adaxial and abaxial sides of leaves, together with NGT and CGT. No trichomes were present on the hypocotyls and cotyledons. In contrast to the CGT, LGT were absent on the anther appendages.

The LGT on stems, leaf petioles, phyllaries and leaves showed a multicellular, uniseriate structure, comprising 6–11 cells and an average length of 165 ± 41 µm (*n* = 30; range 100–266 µm). They emerged from a unicellular basal cell of the epidermis. Except for the usually spherical tip cell, the intermediate cells were barrel shaped to cylindrical (Fig. 1C, G, H and J). In some cases, the tip cell ended conically (Fig. 1I). The number of cells was not related to the age of the leaves. Growth of the trichomes or cell division was not observed, even when very young leaves (2–3 mm in length) from the apical bud were investigated in comparison with fully expanded leaves.

It was conspicuous that LGT on leaves were particularly found along leaf veins. A closer investigation with SEM showed a strict association with the vascular system (Fig. 1J).

The abaxial surface of phyllaries harboured LGT in the highest density. Compared with the leaf petioles, the number of LGT per cm^2^ was ∼3 times higher.

Within the flower head, LGT occurred on the abaxial surface of chaffy bracts. Here, the basal cells had an elongated barrel-shaped form, whereas the tip cells were typically spherical. One to two basal cells showed an accumulation of reddish pigments, which were visible on the chaffs' epidermal cells as well (Fig. 1D). On the abaxial surface of ray and disc florets, the structure of LGT was similar to that on the chaffs, but sometimes the barrel-shaped form of the cells was replaced by cylindrical cells in the front part of the trichome (Fig. 1E and F). The accumulation of yellowish pigments occurred in the basal cells of LGT (and in the NGT), but sometimes also up to the tip cell.

In order to analyse whether new trichomes are formed during leaf expansion, the number of LGT per cm epidermis of the central leaf vein was determined over a period of 12 days. Compared with the trichome density of the first stage (leaf length of primary leaves ∼6 cm), the density of older stages gradually decreased to ∼44 ± 9% within 12 days. At the same time, the length of the central leaf vein had increased by ∼37 ± 21%, indicating that the reduction of the LGT density is a consequence of leaf elongation growth and is not compensated by a substantial generation of new trichomes on the growing tissue.

### Distribution of LGT in related species

Besides *H. annuus*, samples of 40 taxa (see Table 1) of the genus were screened for LGT. All of them showed LGT, although sometimes slightly varying in cell shape, number of cells or colouration of cell content. In *H. glaucophyllus*, a species that generally lacks trichomes on stems and leaves, LGT were located along the veins of the corolla of ray florets.

Within the subtribe Helianthinae (as delineated by Schilling and Panero 2011), LGT were searched for on herbarium specimens of the following genera (number of screened taxa versus total number of genus given in parentheses): *Aldama* (5/118)*, Heliomeris* (4/5)*, Lagascea* (1/1), *Pappobolus* (2/38), *Phoebanthus* (2/2), *Sidneya* (1/2), *Simsia* (2/24), *Tithonia* (4/12) and *Viguiera* (1/1)*.* The occurrence of LGT could be shown in all of the screened genera, except for *Sidneya*; within the different genera, the number of taxa with or without LGT varied and in some cases <10% of the taxa could be screened. In cases where no LGT were observed (e.g. *Sidneya*, some taxa of *Heliomerios* and *Tithonia*), they were absent on plant surfaces that were accessible to microscopy without destroying the specimen. This means that their absence in the inner parts of the flower head (e.g. chaffy bracts, disc florets) needs to be re-analysed in future studies, most adequately with fresh plant material.

Outside of the Helianthinae, LGT were found on *Rudbeckia laciniata*, but too few taxa have yet been screened to provide a reliable overview on a broader distribution, at least in the Heliantheae.

### Metabolic activity of LGT

**Figure 2.**
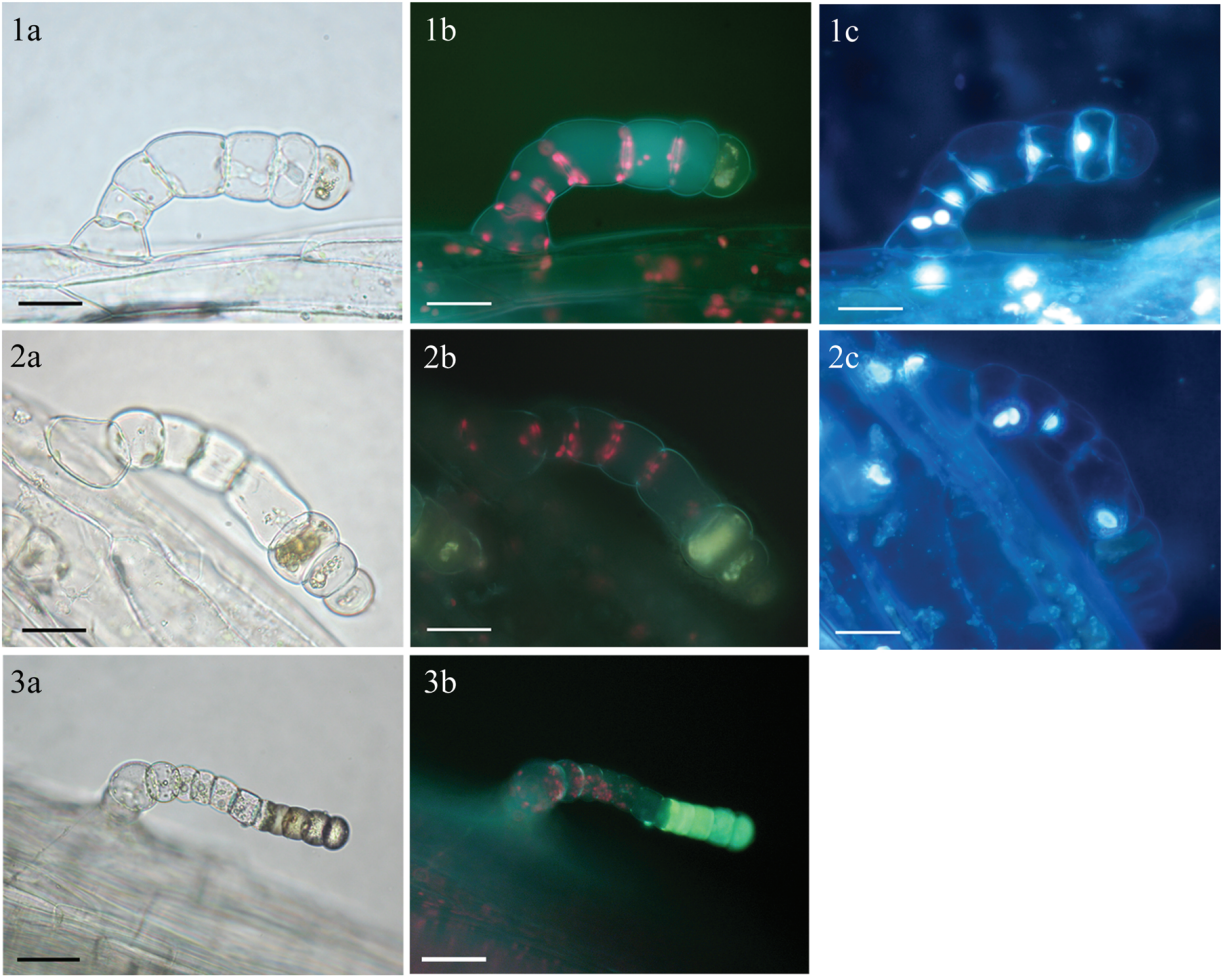
Micrographs of LGT of sunflower. 1a, 2a and 3a: light microscope photographs; 1b, 2b, and 3b: fluorescent microscope photographs (FM, Filter I, 395–440 nm); 3a and 3b: FM photographs (Filter II, G365) after staining with a vital stain (Hoechst 33342). 1 and 2: plants grown in the climate chamber; 3: field-grown plant.

**Figure 3.**
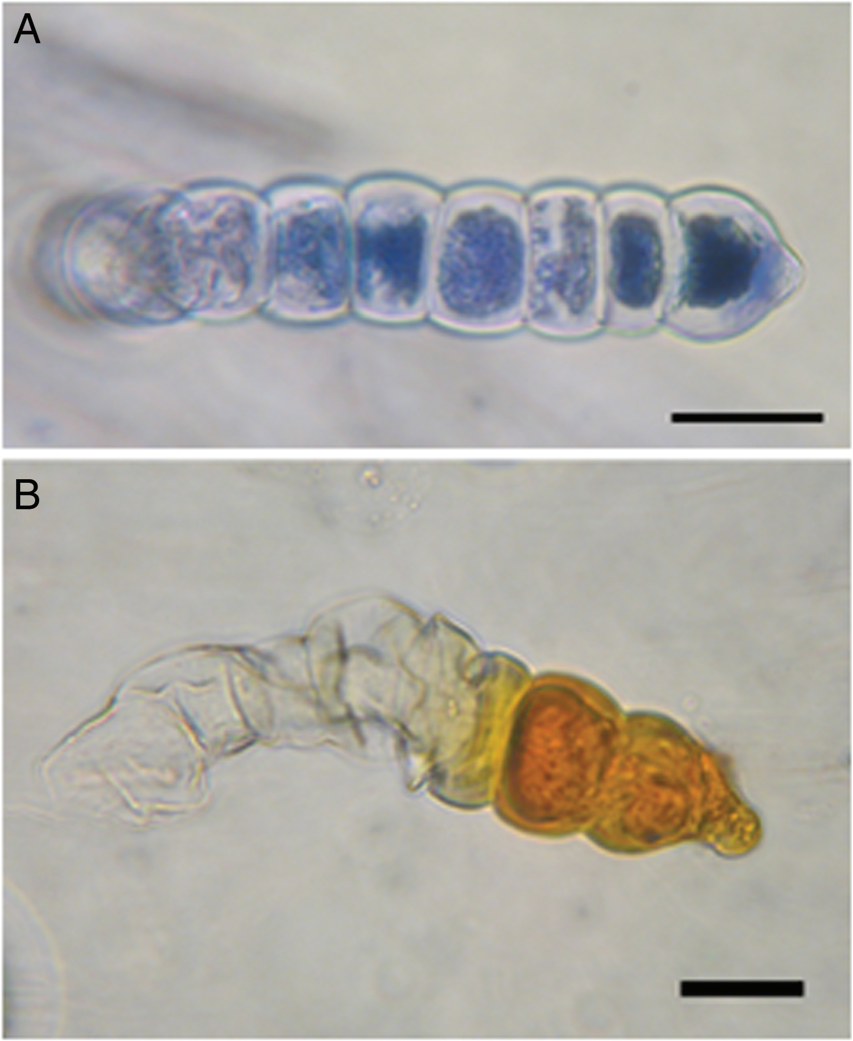
Light micrographs of LGT of sunflower after treatment with Nadi reagent (A) and concentrated H_2_SO_4_ (B). Scale bar: 20 µm.

Microscopic examination (LM and FM) of LGT, on stems, leaf petioles, leaves and phyllaries of sunflower, showed the internal deposition of brownish and fluorescent substances of as yet unknown chemical structure and function (Fig. 2). This accumulation was stronger in the LGT of field-grown plants (Fig. 2, 3a and 3b) when compared with plants grown in the climate chamber (Fig. 2, 1a, 1b, 2a and 2b). Under fluorescent light (395–440 nm, Filter I), the cells that had accumulated these compounds stained yellowish green, whereas the other cells were uncoloured except for the red fluorescence of the chloroplasts (Fig. 2, 1b, 2b and 3b). Staining experiments with the vital stain Hoechst 33342 revealed the presence of nuclei in all cells except the ones filled with the brownish and fluorescent depositions (Fig. 2, 1c, 2c, Filter II: G365). This indicated a loss of vitality that gradually expanded from the tip cell to the basal cells of LGT when the accumulation of the compounds progressed. Staining of LGT with Nadi reagent (Fig. 3A) and concentrated H_2_SO_4_ (Fig. 3B) revealed the presence of terpenes, but the typical blue colour of the Nadi reaction was not limited to the cells containing the green fluorescent compounds visible in Fig. 2. This indicated the different chemical nature of the terpenoid and the fluorescent metabolites.

## Discussion

Only two investigations have so far been published dealing with an LGT type that was firstly found on the leaves of sunflower and was reported to produce sesquiterpenes of the bisabolene type (Spring *et al*. 1992) and to express a different type of sesquiterpene synthase when compared with the capitate trichomes present on the same plant (Göpfert *et al*. 2010). No investigation so far has focused on the trichome morphology and occurrence in sunflower organs or in phylogenetically related taxa. Our study revealed that LGT occurred simultaneously with NGT on almost all plant parts except for anthers and the generally hairless surfaces of the cotyledons and the hypocotyls. Linear glandular trichomes were particularly found on leaf petioles, stems and phyllaries where CGT were absent. While on leaves of sunflower the CGT are usually located in intercostal areas and in small depressions (Spring *et al*. 1987), the LGT are found to be closely associated to the leaf veins. This corroborated our previous findings (Spring *et al*. 1987, 1992) and the localization visible from microscopic plant surface photographs of sunflower leaves published by Harr and Guggenheim (1995). Such an association with the vascular system is not typical for trichomes producing secondary metabolites. Unlike in the case of trichome hydathodes, for which the functional relationship with respect to the location on leaf veins is most obvious (Wagner *et al*. 2004), no such hints are yet known for the occurrence of LGT along vascular bundles.

The morphology of LGT on leaves was rather homo-geneous with respect to the number of cells, their size and shape. Although principally similar in structure, the LGT found on other plant organs showed slight deviations, for example in the average number of cells, or the shape of the intermediate cell (cylindrical instead of barrel shaped). Nevertheless, we assume that all LGT found in this study represent a single uniform type with respect to their structure, physiology and function.

The development of LGT could not be observed in detail, although we attempted to sample leaves at different stages of growth. Obviously, the trichome development in sunflower is completed at a stage where the leaf pri-mordia are still embedded in the plumula. This resembles the early development of CGT reported from sunflower leaves (Spring and Bienert 1987) and the anther appendages of the disc florets (Göpfert *et al*. 2005). A similar situation was reported for trichomes of *Stevia rebaudiana* (Asteraceae) by Monteiro *et al*. (2001), who investigated the early development of CGT on the leaf primordia. Although not mentioned in their paper, a linear type of trichome similar to our LGT is visible on their micrographs from *Stevia* and these trichomes also appear to be fully developed. Our experiments to trace the formation of LGT in older stages of the organogenesis in sunflower showed that their density on the central veins of expanding leaves decreased almost reciprocally to the increase in length of the central leaf vein. This suggests that their total number per leaf is defined early and uninfluenced by external modification. This was in line with the observation that different light regimes during plant cultivation did not alter the density or morphology of LGT observed on leaves (data not shown).

As we have been able to show, the LGT are physiologi-cally intact and show nuclei and functional chloroplasts, as other GT of some Solanaceae and Asteraceae (Cheniclet and Carde, 1985) do. They are metabolically active and produce brownish and fluorescent compounds, which are stored inside the cells. It has been shown that the GT of diverse plant families, such as Lamiaceae, Scrophulariaceae, Asteraceae and Bignoniaceae, accumulate substances in the vacuole or in a subcuticular space (Ventrella and Marinho 2008). However, a solely internal accumulation, without any formation and secretion into a subcu-ticular space, has not been described before.

Light seems to affect the metabolic activity of LGT. It was most obvious that the internal accumulation of brownish and fluorescent compounds accompanied by nucleus degradation was significantly stronger and involved more cells when plants were cultivated outdoors in sunlight instead of in the climate chamber (see Fig. 2, 3a and 3b). Similar light effects on the trichome metabolism have been previously reported from the CGT of sunflower, where the formation of STL was triggered by the phytochrome system (Spring and Bienert 1987) and the compound pattern was altered by the light quality during plant cultivation (Spring *et al*. 1989).

For the LGT, additional phytochemical studies are required to understand the evolutionary, environmental and functional aspects of their metabolic activity. So far, only bisabolene-type sesquiterpenes have been identified (Spring *et al*. 1992). Due to their volatility, these bisabolenes may account for the chemo-ecological interaction of sunflower with pollinators or herbivores, as was shown for other phytochemicals deposited on sensitive plant organs (for references see Wagner *et al*. 2004). On the other hand, the sesquiterpenes do not account for the fluorescence found in sunflower LGT, thus indicating the presence of additional metabolites of other substance classes. It is also completely unknown whether the LGT found in other plant species contain the same compounds found in *H. annuus* (Spring *et al*. 1992). The occurrence of LGT in a broad spectrum of taxa screened during this study underlines their functional importance. It may also provide additional phylogenetic character, at least for the Helianthinae, but perhaps even at the tribe level of the Heliantheae (e.g. *Rudbeckia*) and the Eupatorieae (e.g. *Stevia*).

## Conclusions

Sunflower and related taxa of the Helianthinae possess a type of multicellular, uniseriate, metabolically active trichome that has not been reported from other tribes of the Asteraceae before. The localization of these trichomes on prominent plant parts of the apical bud and the capitulum combined with the accumulation of terpenoids and other as yet unknown compounds suggests a chemo-ecological function of the LGT in the plant–insect or plant–herbivore interaction.

## Sources of Funding

This work was supported by the Deutsche Forschungsgemeinschaft (DFG, German Research Foundation; project SP292/22-1).

## Contributions by the Authors

A.-K.A. performed all major experiments and contributed to manuscript preparation. S.H. made major contributions with respect to the occurrence of LGT in related species. O.S. planned and coordinated the project and contributed to manuscript preparation.

## Conflicts of Interest Statement

None declared.
